# Towards multimodal graph neural networks for surgical instrument anticipation

**DOI:** 10.1007/s11548-024-03226-8

**Published:** 2024-07-10

**Authors:** Lars Wagner, Dennis N. Schneider, Leon Mayer, Alissa Jell, Carolin Müller, Alexander Lenz, Alois Knoll, Dirk Wilhelm

**Affiliations:** 1 Technical University of Munich, TUM School of Medicine and Health, Klinikum rechts der Isar, Research Group MITI, Munich, Germany; 2 Technical University of Munich, TUM School of Medicine and Health, Klinikum rechts der Isar, Department of Surgery, Munich, Germany; 3 Technical University of Munich, TUM School of Computation, Information and Technology, Chair of Robotics, Artificial Intelligence and Real-Time Systems, Munich, Germany

**Keywords:** Surgical data science, Graph neural networks, Surgical instrument anticipation, Surgical process modeling

## Abstract

**Purpose:**

Decision support systems and context-aware assistance in the operating room have emerged as the key clinical applications supporting surgeons in their daily work and are generally based on single modalities. The model- and knowledge-based integration of multimodal data as a basis for decision support systems that can dynamically adapt to the surgical workflow has not yet been established. Therefore, we propose a knowledge-enhanced method for fusing multimodal data for anticipation tasks.

**Methods:**

We developed a holistic, multimodal graph-based approach combining imaging and non-imaging information in a knowledge graph representing the intraoperative scene of a surgery. Node and edge features of the knowledge graph are extracted from suitable data sources in the operating room using machine learning. A spatiotemporal graph neural network architecture subsequently allows for interpretation of relational and temporal patterns within the knowledge graph. We apply our approach to the downstream task of instrument anticipation while presenting a suitable modeling and evaluation strategy for this task.

**Results:**

Our approach achieves an F1 score of 66.86% in terms of instrument anticipation, allowing for a seamless surgical workflow and adding a valuable impact for surgical decision support systems. A resting recall of 63.33% indicates the non-prematurity of the anticipations.

**Conclusion:**

This work shows how multimodal data can be combined with the topological properties of an operating room in a graph-based approach. Our multimodal graph architecture serves as a basis for context-sensitive decision support systems in laparoscopic surgery considering a comprehensive intraoperative operating scene.

## Introduction

**Fig. 1:**
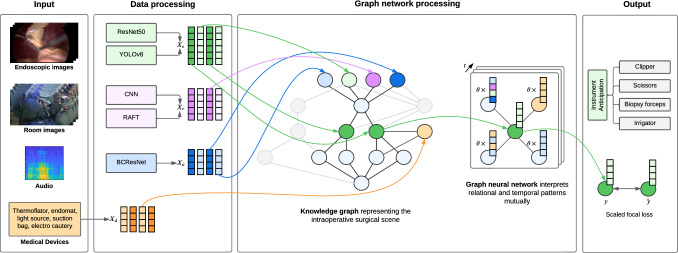
Overview of our multimodal surgical workflow anticipation pipeline using graph neural networks. The different data modalities are preprocessed in unimodal architectures. The output of the unimodal models ($X_e, X_r, X_a, X_d$) forms the node embeddings of the knowledge graph. These embeddings are then refined in a spatiotemporal graph neural network to predict the most probable outcome *y*. The comparison to the ground truth $\hat{y}$ is facilitated by a scaled multi-class focal loss function. As part of context-sensitive decision support systems, we anticipate the surgeon’s needs by predicting the next required instrument. During this task, we exclude the embedding of audio data aiming for non-verbal communication during instrument handovers

In the field of surgical data science (SDS), artificial intelligence (AI)-based decision support and context-aware assistance systems have emerged as the key clinical applications supporting surgeons in their daily work while improving patient outcomes [1]. Particularly in the operating room (OR), such systems can provide surgeons with real-time information to augment surgical decision-making [2]. To enable situation-dependent decisions in the OR, the integration of multimodal data is essential. Incorporating multimodal signals, such as the patient’s vital signs, imaging data such as the endoscopic image or medical device data, provide the basis for decision support systems that adapt dynamically to the surgical workflow. However, a comprehensive combination of these multimodal data sources providing a quantitative description of the surgical state has not yet been established [3]. The reason for this is the large scale and velocity of the multimodal data generated from heterogeneous sources of raw data [1] and the difficulty in accessing this data [4], leading to assistance systems integrating only one data source.

Remedy can be provided by graph neural networks (GNNs) offering an expressive and flexible strategy to leverage interdependencies in multimodal environments and embedding contextual knowledge [5]. They are used to learn representations of different graph components such as nodes, edges, or entire graphs based on message passing strategies [6]. Typical applications of GNNs are node prediction, link prediction, or community detection tasks [7]. In the area of SDS, GNNs have so far only been applied to the *internal surgical scene* for tasks like surgical phase recognition [8], tool presence detection [9], or recognizing instrument-action-tissue triplets [10]. In contrast, graph-related analysis of the *external surgical scene* has been mainly performed by neural network-based scene graph generations for tasks such as clinical role prediction or surgical phase recognition [11]. The generated scene graph models the numerous participants, objects, and their interactions, viewing the operating room as a singular, interconnected entity rather than a collection of distinct activities. To provide a holistic view of the surgical workflow, the *internal* and *external surgical scene* need to be merged.

**Fig. 2:**
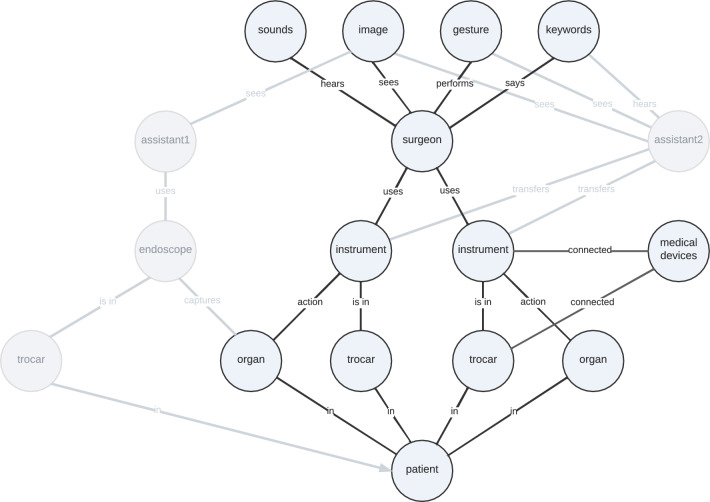
Knowledge graph of the external and internal surgical scene representing the intraoperative state. The section of the graph considered in this work is shown in blue. The graph can be extended modularly by adding further entities and relationships

Therefore, we introduce a static knowledge graph that combines the entities and relationships from the *internal* and *external surgical scene* representing the intraoperative state. We incorporate multiple data modalities such as image and medical device data by using several backbone models which process the individual modalities and pass their outputs to the knowledge graph, resulting in a modular joint fusion [12] architecture. The graph serves as a foundation for context-sensitive decision support systems in laparoscopic surgery and is learned in this paper by means of GNN on the downstream task of instrument prediction (Fig. 1). The main contributions of this paper are:

- We present a modular knowledge graph which represents the intraoperative state of a surgery.
- We demonstrate how downstream tasks such as surgical instrument anticipation can be realized on the basis of the knowledge graph using GNNs.
- We conduct ablation studies and thus highlighting the importance of individual data sources.

## Methods

### Graph modeling

A structured representation of the surgical process is a prerequisite for any context-sensitive application in the OR [13]. A variety of surgical workflow modeling approaches exist [14], such as statistical and machine learning methods [15] or ontology-based approaches [13]. However, since the surgical process can be divided into several granularities of time frames, we do not model the entire temporal sequence of a laparoscopic surgery, but first provide a static low-level representation of the intraoperative scene using a knowledge graph with a set of nodes and a set of edges (Fig. 2). The entities and relations of the graph were derived from the surgomics approach in [3]. The nodes and edges represent the participants and their interactions with the *internal* and *external* surgical environment which are automatically extracted from suitable data sources using machine learning. By updating these features in real time, we obtain both the spatial and temporal representation of the surgical process. In this paper, we focus on the surgeon and their interactions with patients, as well as the perceptions they gain from the operating suite.

### Data sets

We used three different in-house data sets to train both the backbone models (node models) and the GNN (graph model). The first data set $S_1$ consists of 50 laparoscopic videos of cholecystectomies including binary annotations of instruments, actions, and phases. The second data set $S_2$ consists of 12 laparoscopic videos of cholecystectomies providing pixel-wise annotations of instruments and anatomical structures. The third data set $S_3$ provides time-synchronized recordings of 10 cholecystectomies of the laparoscopic video, room video, audio, and medical devices with annotations of the instruments and their interactions with the anatomical structure resembling the instrument, verb, target annotation strategy in [16]. The data set also contains the performed gestures, which are presented in section “Node models”. The annotations for all data sets were evaluated by medical experts. However, since the annotation of a multimodal data set with medical expertise is very expensive [4], we reduce the prediction task to the clipping phase. Table 1 shows the number of frames of the target classes in the training and validation data set.

**Table 1:** Number of frames of the target classes for the training and validation data set

Class	Training set	Validation set
Idle	27,368	5249
Clipper	276	102
Scissors	102	36
Biopsy forceps	66	24
Irrigator	18	0

**Fig. 3:**
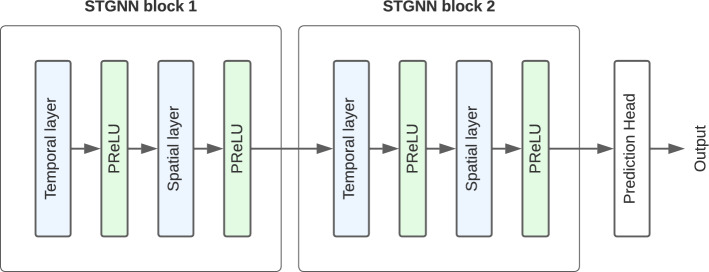
Schematic architecture of our network which is composed of two STGNN blocks. Each block comprises a temporal layer and a spatial layer, both followed by PReLU activation functions. The output of the second block is passed through a prediction head and provides the final model output

### Network architecture

As illustrated in Fig. 1, there are two data sources (endoscopic image and room image), which are preprocessed by separate unimodal models. The outputs of these models constitute the embeddings of the nodes *image*, and *gesture* being refined by a spatiotemporal GNN to predict the next required instrument. The medical device data forms directly the node embeddings of the node *medical devices* and is not preprocessed. The following section briefly introduces the individual node models, while the architecture of the graph model is explained afterwards.

#### Node models

*Endoscopic image features*: The features of the endoscopic image are extracted using a ResNet50 [17] and a YOLOv8 [18] architecture. The ResNet50 was trained frame-wise without temporal context on the basis of data set $S_1$ for the detection of instruments and actions. The YOLOv8 was trained on the basis of data set $S_2$ also for instrument detection as well as for organ and instrument segmentation. From the segmentation masks, different image shape measurements were calculated for each detected organ and instrument. Assuming that the second last layer of the ResNet50 contains more information than the output probabilities, we concatenate the second last layer with the class output probabilities and the outputs of the YOLOv8 network.

*Room image features*: The room image features are extracted using a custom CNN and the optical flow-based model RAFT [19]. The room image shows the *external* surgical scene, allowing for inference of surgical phases based on the surgeon’s movements. The models were trained to recognize these phases based on the data set $S_3$. The phases include operating, extraction of the instrument, handover of the instrument with the scrub nurse, and insertion of the instrument. A skip connection from the penultimate layer to the last layer provides enrichment of the model’s feature representations.

**Fig. 4:**
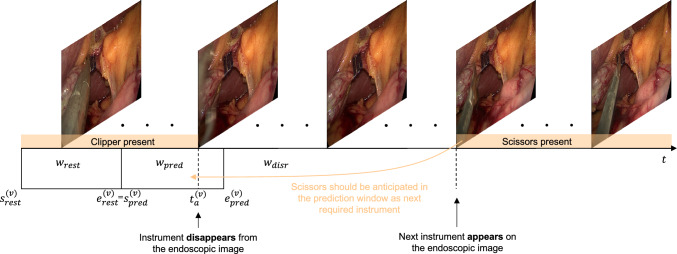
The time point $t_a^{(v)}$ denotes the anchor point of the *v*-th instrument change, with $s_\textrm{pred}^{(v)}$ and $e_\textrm{pred}^{(v)}$ is the start- and end times of the prediction window $w_\textrm{pred}$. The resting window $w_\textrm{rest}$ is delimited by analogous boundaries. Predictions not falling into either a prediction or resting window will belong to a disregard window $w_\textrm{disr}$ in which predictions do not harm the crucial workflow of the surgeon. The figure exemplifies the extraction of the clipper while the scissors is to be anticipated

#### Graph models

The heterogeneous setting of the knowledge graph has a major influence on the design of the graph model architecture. Considering the unique characteristics of the knowledge graph, we develop a generic spatiotemporal GNN (STGNN) architecture allowing for suitable and flexible selection of different temporal and spatial layers. Due to the small size of our graph compared to natural graph problems, we only stack two GNN layers to avoid overlapping of receptive fields of individual nodes and thus prevent oversmoothing problems [20]. As shown in [21], prediction tasks on static graphs benefit from first processing time-series features before performing graph representation learning. This motivates the architecture presented in Fig. 3. The spatial layer is based on the general design space for GNNs proposed by [22]. Our generic architecture includes a PReLU activation function [22] after each layer introducing an additional learnable parameter. In the following, we present suitable temporal and spatial components, whose combination distills both spatial information about the neighborhood of a node and its state over time.

*Temporal components*: The temporal part of the architecture can be modeled by incorporating a multitude of sequential processing models. Therefore, we use the ones most prevalent in current research in the field of graph machine learning. These include the recurrent architectures LSTM [23] and GRU [24], which both utilize gating mechanisms to manage information flow and address the vanishing gradient problem. Alternatively to the sequential processing models of recurrent nature, we also investigate the behavior of convolutional models, such as the dilated temporal convolution (DTC) architecture.

*Spatial components*: To handle the heterogeneity of the knowledge graph, we project the feature information of each node and edge type into a common space of same dimensionality. Multiple works follow a similar scheme to interrelate neighboring nodes of multiple node types [25]. We generalize the approaches *GraphSAGE* [26], *HEAT* [27], and *AGNN* to the heterogeneous domain and expand them by incorporating edge features. Edge attributes are included by concatenation with neighboring node attributes as in [28]. The adaptions are referred to as heterogeneous *EiSAGE* (Edge induced SAGE), *HEATv2*, and *AGNNv2* below.

*Prediction head*: We handle the task of instrument anticipation as a multitask approach with two classification heads. The first prediction head is responsible for classifying the next required instrument, while the second prediction head is responsible for recognizing an instrument change. The recognition task of an instrument change serves as a gating mechanism for the instrument prediction task.

### Task formulation

To provide OR assistants with the information about the next required instrument so that the surgeon receives the instrument in time, a certain prediction window $w_\textrm{pred}$ has to be established, in which the instrument prediction process should take place. For this purpose, we anchor the end of the prediction window $e_\textrm{pred}^{(v)}$ on the time of instrument extraction of the *v*-th instrument change. Instrument extraction refers to the disappearance of the instrument from the endoscopic image and the subsequent removal of the instrument by the surgeon from the trocar. The reasoning behind this approach is the deterministic behavior of the surgeon, who extracts the instrument after being finished with his current action. To allow for instrument preparation by the assistant, the prediction of the next instrument should align with the start of the prediction window, rather than coinciding with the extraction of the previous instrument at the end of the window. However, premature prediction should not take place, which is why we introduce a resting window $w_\textrm{rest}$, which precedes the prediction window. In this window, no instrument should be anticipated as the surgeon is not yet close to completing the surgical action. Additionally, the prediction window is shifted to overlap the point of instrument extraction, allowing the model to learn on disappearance of the instrument from the endoscopic image. Figure 4 visualizes the prediction and resting window related to the extraction time.

### Experimental setup

#### Model training

The STGNN was trained on data set $S_3$ using the Adam optimizer with an initial learning rate of $1e-4$ for 40 epochs. The 10 video sequences of the clipping phase were randomly split into training ($70\%$) and validation ($30\%$) set. The batch size was 600. For the prediction window boundaries we selected $s_\textrm{pred}^{(v)} = t_a^{(v)} -4s$ and $e_\textrm{pred}^{(v)} = t_a^{(v)} +2s$, while for the resting window boundaries we selected $s_\textrm{rest}^{(v)} = t_a^{(v)} -12s$ and $e_\textrm{rest}^{(v)} = t_a^{(v)} -4s$. We used a scaled multi-class focal loss [29] function for joint training of the model. We provide the results of the model that performed best on the validation set. The model was implemented in PyTorch and trained on three NVIDIA RTX A6000s.

#### Evaluation metrics

To assess the performance of our proposed method, we report mean-averaged metrics such as precision, recall, accuracy, and F1-score for the node models. We report the weighted-averaged F1-score (wAF1) and micro-averaged recall (mAR) for the graph models to measure anticipative performance on the one hand and to assess the prematurity of the predictions on the other. In weighted averaging, each metric is scaled according to its sample distribution in the test data set. A prediction is considered successful if at least nine of the last ten predictions made by the model are correct.

## Results

We present the results of the individual node models, followed by the results of the STGNN with varying temporal and spatial layers.

### Node models

*Endoscopic image*: In Table 2, we show the results of the surgical instrument and action recognition of the ResNet50 feature extraction backbone for the instruments and actions relevant in the clipping phase. While the instruments clipper (F1 $89.80 \%$), biopsy forceps (F1 $89.96 \%$) and irrigator (F1 $87.46 \%$) show high F1 scores, these drop slightly for the grasper and scissors. The dissection (F1 $83.93 \%$), cutting (F1 $81.49 \%$), and clipping (F1 $83.45 \%$) actions all provide F1 scores above the threshold of $80 \%$. The cleaning action achieves a lower F1 score (F1 $71.63 \%$).

**Table 2:** Mean average precision, recall, accuracy, and F1 score for surgical instrument and action recognition

Instrument	Precision	Recall	Accuracy	F1
Grasper	87.57	77.42	93.03	81.22
Clipper	95.31	92.33	99.35	89.80
Scissors	85.32	68.04	99.49	79.85
Biopsy forceps	95.97	85.70	93.99	89.96
Irrigator	93.89	85.00	96.25	87.46
Action				
Dissection	93.20	75.56	95.12	83.93
Cleaning	82.81	62.09	95.28	71.63
Cutting	90.17	79.43	96.69	81.49
Clipping	90.33	84.45	95.25	83.45

The averaged metrics of predictions relevant for the clipping phase are reported (%)

**Table 3:** Mean average precision, recall, accuracy, and F1 score for surgical gesture recognition

Gesture	Precision	Recall	Accuracy	F1
Operating	95.28	83.55	87.62	89.03
Extraction	41.22	40.68	91.74	40.95
Handover	55.74	59.22	82.06	57.43
Insertion	83.68	47.39	92.33	60.51

The averaged metrics of the four gesture phases are reported (%)

**Fig. 5:**
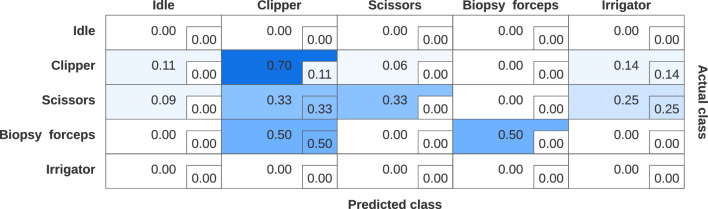
Multi-class confusion matrix of anticipated surgical instruments over 5 independent runs. The matrix compares the predicted classes (clipper, scissors, biopsy forceps, irrigator) against the actual classes. Each cell shows the row-wised normalized proportion of predictions, with values ranging from 0 to 1, showing the recall for each instrument. The embedded numbers in each cell represent the proportion of premature predictions

*Room image*: The combination of a CNN and RAFT achieves the results presented in Table 3 for the detection of *external* surgical phases. The operating phase (F1 $89.03\%$) is recognized best. The insertion phase and the handover phase achieve an F1 score of $60.51\%$ and $57.43\%$, while the extraction phase (F1 $40.95\%$) phase yields a lower score, indicating that these phases are challenging to recognize without including temporal information.

**Table 4:** Weighted-averaged F1 score (first row) and micro-averaged recall (second row) of the STGNN architecture for the task of surgical instrument anticipation

Spatial layer	Temporal layer		
	LSTM	GRU	DTC
EISAGE	$57.52 \pm 4.68$	$51.14 \pm 5.12$	$\mathbf {66.86} \pm \mathbf {3.94}$
	$43.67 \pm 4.72$	$40.71 \pm 4.31$	$\mathbf {63.33} \pm \mathbf {8.61}$
HEATv2	$42.21 \pm 4.34$	$50.38 \pm 6.96$	$52.53 \pm 5.50$
	$40.00 \pm 7.43$	$53.33 \pm 10.30$	$47.43 \pm 6.17$
AGNN	$47.85 \pm 8.12$	$45.20 \pm 6.17$	$49.10 \pm 7.52$
	$45.81 \pm 7.15$	$45.20 \pm 7.95$	$48.35 \pm 7.25$

The best mean values are highlighted in bold

The performance of different combinations of spatial and temporal layers is compared. The averaged metrics over 5 independent runs are reported (%) with the corresponding standard deviation (±)

### Graph models

#### Instrument anticipation

For the task of instrument anticipation, we compare the performance of different spatial and temporal layers within our STGNN architecture. We report the weighted-averaged F1 score (wAF1) for all our approaches considering the number of occurrences of each class in the test data set. In addition, we also provide the micro-averaged recall (mAR) of the resting window, indicating premature prediction of the respective instrument.

As shown in Table  4, the best result is achieved with EiSAGE as spatial layer and DTC as temporal layer with a wAF1 score of $66.86\%$. Thus, our best architecture is able to predict $66.86\%$ of instrument changes correctly. The corresponding wAR of $63.33\%$ indicates the proportion of predictions that are actually made in the prediction window. It can be seen that a specific proportion of predictions is already made in the resting window. Figure 5 shows the multi-class confusion matrix of the instruments relevant for the clipping phase. The matrix reveals varying levels of classification performance across the four different instruments. The model exhibits a strong predictive accuracy for the clipper class with a probability of 0.70, followed by a moderate accuracy for scissors and biopsy forceps with probabilities of 0.33 and 0.50, respectively.

#### Ablation studies

We conducted ablation studies to measure the impact of the different nodes of the knowledge graph in terms of the anticipation task. For this purpose, we removed individual nodes while learning the best model from section “Instrument anticipation”. In Table  5, we report the changed model performance in terms of wAF1. The lowest wAF1 score is achieved by ablating the node *image* indicating that the node model learned on the endoscopic image extracts the most important information for the prediction. Excluding node *gesture* provides a smaller impact on the model performance; however, this has a stronger effect on the mAR, which decreases to $45.25\%$. Ablating the node *medical devices* provides the smallest drop in both the waF1 and mAR score.

**Table 5:** Weighted-averaged F1 score (wAF1) and micro-averaged recall (mAR) as a result of removing individual nodes

Ablative node	wAF1	mAR
Image	$53.62 \pm 3.05$	$55.68 \pm 5.71$
Gesture	$60.81 \pm 6.07$	$45.25 \pm 3.69$
Medical devices	$64.18 \pm 7.82$	$57.82 \pm 5.10$

The score over 5 independent runs is reported (%) with the corresponding standard deviation (±)

## Discussion

Our work contributes substantially to the field of SDS by introducing a knowledge enhanced method for fusing multimodal OR data for anticipation tasks. We propose a modular architecture in which the individual node models, the knowledge graph and the graph model can be easily extended or exchanged. The node models act as feature extractor backbones, providing the node embeddings of the knowledge graph. Using the knowledge graph, we exploit the topological properties of the operating theater to enable selective information flows in the graph model. We introduce a generic STGNN architecture that leverages the node embeddings of the knowledge graph to capture and model both spatial and temporal dependencies, thereby enhancing the ability to identify and predict complex patterns and relationships within the graph. We thereby extend existing spatial layers available in the literature with the handling of heterogeneity and edge features.

Context-sensitive decision support systems, such as instrument anticipation, have great potential for various applications in the surgical environment. They do not only aid operating room assistants in the desired seamless transfer of surgical instruments to the surgeon but also serve as valuable training tool for novices to maintain the surgical workflow. However, the results show that not all instruments can be accurately predicted. This is mainly due to the very dynamic instrument changes by the different surgeons, performing the operations according to different standards. In addition, the exclusive focus on the clipping phase, which is characterized by action-related instrument, changes limits the overall performance of the approach. Considering entire surgical sequences of cholecystectomies with typically phase-related instrument changes, our approach would achieve an overall better performance. Therefore, the future focus will be on increasing the annotated data set to learn the patterns of both action- and phase-related instrument changes as well as the investigation of a regression head instead of a gating mechanism, as regression approaches are very suitable for determining temporal boundaries of instrument occurrences [30–32]. In addition, enlarging the data set should also ensure a more balanced training and validation set. Since the validation set does not include the occurrence of the irrigator, it is difficult to ensure that the model generalizes well to new irrigator instances. This imbalance can also bias the model toward other classes, potentially reducing its sensitivity to the irrigator class, which we are currently trying to prevent by class weighting of the loss function during training.

As the current implementation state does not yet allow for real-time inference, it is also necessary to develop strategies for real-time deployment in the OR. Besides model optimization through techniques such as quantization, layer and tensor fusion, and kernel tuning, our strategy will pivot toward implementing a decentralized computing framework, where modalities are first processed on separate computing units before being combined, targeting a hybrid or late fusion approach.

There are additional ways to improve the performance of the model, such as incorporating more reliable feature extractors or considering dependencies between individual modalities during feature extraction. However, this approach is a first step toward a model- and knowledge-based incorporation of multimodal data for the task of instrument anticipation, which provides promising results even on a small amount of data.

## Conclusion

We propose a knowledge enhanced method for fusing multimodal OR data for anticipation tasks. Therefore, we present a possible modeling and evaluation strategy for the task of instrument change prediction. Our modular architecture achieves an F1 score of $66.86\%$ in terms of instrument prediction. In the future, we want to expand our approach to include additional nodes and relationships as well as further node models in order to provide an even more accurate representation of the intraoperative OR scene serving as a foundation for context-sensitive decision support systems in laparoscopic surgery.

## Data Availability

The data that support the findings of this study are available on request from the corresponding author. The data sets are not publicly available due to restrictions related to privacy concerns for the research participants.
